# Solid-State Fermentation of Cereal Waste Improves the Bioavailability and Yield of Bacterial Cellulose Production by a *Novacetimonas* sp. Isolate

**DOI:** 10.3390/foods13193052

**Published:** 2024-09-25

**Authors:** Shriya Henry, Sushil Dhital, Huseyin Sumer, Vito Butardo

**Affiliations:** 1 Department of Chemistry and Biotechnology, Swinburne University of Technology, Hawthorn, Melbourne, VIC 3122, Australia; shriyahenry@swin.edu.au (S.H.); hsumer@swin.edu.au (H.S.); 2 Department of Chemical and Biological Engineering, Faculty of Engineering, Monash University, Clayton, VIC 3800, Australia; sushil.dhital@monash.edu

**Keywords:** bacterial cellulose, biopolymer, solid-state fermentation, rice bran, cereal dust, cereal waste, *Novacetimonas*

## Abstract

Cereal wastes such as rice bran and cereal dust are valuable yet underutilised by-products of grain processing. This study aimed to bio-convert these wastes into bacterial cellulose (BC), an emerging sustainable and renewable biomaterial, via an inexpensive solid-state fermentation (SSF) pre-treatment using three mould isolates. Medium substitution by directly using untreated rice bran or cereal dust did not significantly increase the yield of bacterial cellulose produced by *Novacetimonas* sp. (NCBI accession number PP421219) compared to the standard Hestrin–Schramm (HS) medium. In contrast, rice bran fermented with *Rhizopus oligosporus* yielded the highest bacterial cellulose (1.55 ± 0.6 g/L dry weight) compared to the untreated control (0.45 ± 0.1 g/L dry weight), demonstrating an up to 22% increase in yield. Using the SSF process, the media production costs were reduced by up to 90% compared to the standard HS medium. Physicochemical characterisation using SEM, EDS, FTIR, XPS, XRD, and TGA was performed to gain insights into the internal structure, morphology, and chemical bonding of differently produced BC, which revealed comparable biopolymer properties between BC produced in standard and waste-based media. Hence, our findings demonstrate the effectiveness of fungal SSF for transforming abundant cereal waste into BC, providing a circular economy solution to reduce waste and convert it into by-products to enhance the sustainability of the cereal industry.

## 1. Introduction

Bacterial cellulose (BC) is an emerging biopolymer with unique properties such as a high purity, crystallinity, and mechanical strength, making it superior to plant-derived cellulose for various applications [1,2]. Various genera within acetic acid bacteria (AAB), including *Gluconacetobacter*, *Acetobacter*, and *Komagateibacter*, are known for their ability to produce cellulose. *Acetobacter*, commonly found in vinegar, is a heterotrophic bacteria capable of converting glucose, glycerol, sugars, and other organic compounds into cellulose [3]. Although several bacteria that generate bacterial cellulose have been identified, much research has concentrated on model organisms like *Gluconacetobacter xylinus.* This species is particularly notable for its ability to utilise a wide variety of carbohydrates, making it the most important cellulose-producing bacteria [4].

BC and its derivatives are in high demand due to their extensive commercial applications across various industries, such as biomedical, pharmaceutical, cosmetics, packaging, electronics, and food. The global market for BC is projected to expand at a compound annual growth rate (CAGR) of 12.6% from 2019 to 2027, potentially reaching USD 771.76 million. With an estimated annual production of 1012 tons of this biopolymer, the strong market demand ensures a consistent supply of renewable and biodegradable BC raw materials [5]. However, the scaling of industrial BC manufacturing remains restricted by the high costs of conventional culture media [2]. Solid-state fermentation (SSF) has emerged as a promising strategy to enhance the nutritional potential of carbohydrate-rich waste streams, offering advantages such as a reduced reactor volume, lower energy requirements, and simplified downstream processing [6].

Rice bran and cereal dust, abundant waste of grain processing, represent promising yet underutilised substrates for BC production. Annually, rice bran production exceeds 29 million tonnes globally, while cereal dust constitutes approximately 2–3% of grain weight during milling [7,8]. These materials are rich in complex carbohydrates, proteins, and essential nutrients, potentially supporting microbial growth and metabolism [9,10,11]. Previous studies have demonstrated the feasibility of using rice bran as an alternative nitrogen source for BC production, with yields increasing up to 2.4-fold compared to the standard medium [8]. Substrates such as cereal grains have been explored, but there is a noticeable gap in the existing literature regarding the use of cereal dust specifically to produce bacterial cellulose. Limited research has been conducted in this area, making this study particularly novel and impactful. However, the fibrous architecture of these residues can limit nutrient accessibility without preprocessing, and to address that, SSF was used as an approach.

SSF has emerged as an optimal bioprocessing strategy to enhance the nutritional and functional potential of carbohydrate-rich waste streams from cereal milling and rice production [12]. SSF offers advantages such as being suitable for tiny effluent amounts and simple substrates, being cost-effective, and having no extra considerations for standard procedure control. This technique is usually applied to agro-industrial waste resources [13]. SSF utilises filamentous fungi perfectly adapted to solid substrates in nature to break down complex-matrix polysaccharides through secreted enzyme cocktails into readily assimilable sugars, proteins, and metabolites. *Aspergillus niger*, *A. oryzae*, *A. parasiticus*, *Acremonium crysogenum*, and *Rhizopus oryzae* are some of the microorganisms which have been explored for the SSF of rice bran, wheat bran, and other agricultural wastes and by-products [13,14,15,16,17,18]. The literature confirms that SSF significantly increased the protein accessibility of rice bran by unlocking nutrients entrapped within fibrillar networks using natural enzymatic biocatalysts [19].

SSF is particularly advantageous for bioprocessing agro-industrial waste streams, as it operates with a low moisture content (typically < 70%), making it well-suited for materials with low water activity, such as lignocellulosic biomass. The technique offers several economic and sustainability benefits, including a reduced reactor volume, lower energy requirements for aeration and mixing, and simplified downstream processing compared to submerged fermentation. SSF also allows for the direct use of unfractionated solid substrates, minimising the need for costly pre-treatment steps. Moreover, the low water activity in SSF helps prevent bacterial contamination, allowing for a semi-sterile process without the need for strict aseptic conditions. These advantages have made SSF an attractive bioprocessing strategy for the valorisation of agro-industrial wastes into value-added products like enzymes, organic acids, and biofuels [12,13,17,20].

**Figure 1:**
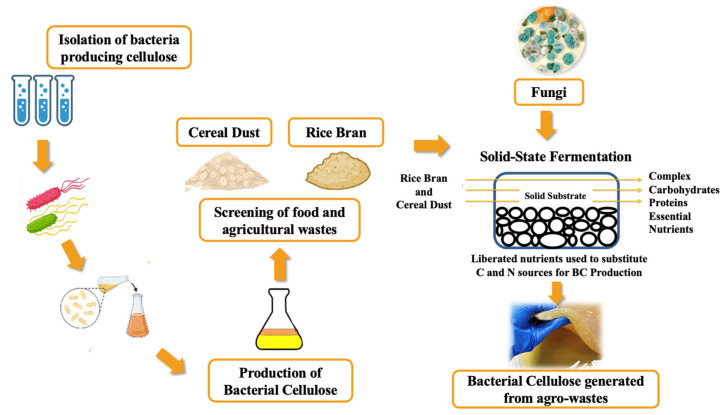
Summarised representation of bacterial cellulose production from solid-state fermented cereal wastes [21].

To address the challenge of producing BC sustainably and at a reduced cost, this study aimed to develop an SSF-based bioprocess for valorising rice bran and cereal dust into BC using three fungal strains: *Rhizopus oryzae*, *Pleurotus ostreatus*, and *Rhizopus oligosporus*. The specific objectives were to (a) assess the impact of SSF pre-treatment on nutrient bioavailability and BC production potential, (b) compare the yield and physicochemical properties of BC produced from untreated and SSF-treated waste streams, and (c) characterise the resulting BC materials using a suite of analytical techniques such as SEM, EDS, FTIR, XRD, and TGA. Additionally, we conducted a preliminary cost analysis to assess the economic feasibility of our approach. By addressing these objectives, this study provides a proof-of-concept for the sustainable valorisation of agro-food wastes into high-value BC biomaterials, potentially paving the way for cost-effective and eco-friendly BC production processes (Figure 1).

## 2. Materials and Methods

### 2.1. Microorganisms and Preparation of Inoculum

A novel *Novacetimonas* sp., an acetic acid bacterium isolated from apple cider vinegar, was used in this study [21]. The isolation process involved screening multiple fermented liquid samples by subculturing them in Hestrin–Schramm (HS) medium to test for consistent bacterial cellulose pellicle formation. This isolate from apple cider vinegar demonstrated stable BC production in repeated subcultures. An initial characterisation through biochemical testing revealed that this isolate belongs to the acetic acid bacteria group. The genomic DNA extraction was carried out using an in-house buffer solution containing Tris-HCl, EDTA, and SDS. Genomic DNA samples were amplified using 27F: 5′-AGAGTTTGATCCTGGCTCAG-3′ and 1492R: 5′-TACGGTTACCTTGTTACGACTT-3′ 16S rRNA universal primers [22]. Further identification was performed using 16S rRNA gene sequencing, which identified that the isolate belongs to the *Novacetimonas* genus with a 99% similarity to known sequences. The 16S rRNA sequence was submitted to NCBI with the accession number PP421219. Gram staining and electron microscopy illustrated the rod-shaped morphology characteristic of this species [21]. The growth profile analysis exhibited an extended exponential phase, faster cell growth in agitated cultures, and higher BC production in static cultures [21]. This isolate represents a promising candidate for BC production due to its robust growth and cellulose-synthesising capabilities. Comparative genomic analyses are currently ongoing to identify this novel isolate down to the species level and to characterise its BC biosynthetic and secretion pathways.

The isolate was grown in HS culture medium for two weeks at 28 °C in a Thermoline Refrigerated Incubator (Model Number TRISLH-495-1-SD, Sydney, Australia) by the static cultivation method to prepare the inoculum. After the incubation period, the flask used to prepare the inoculum was shaken vigorously each time to let the bacterial cells detach from the BC pellicle and mix with the HS inoculate culture medium. This cell suspension, adjusted to 0.1 OD, served as the inoculum. A 10% (*v*/*v*) inoculum concentration was used for all experiments.

### 2.2. Waste Materials Used for the Reformulation of Bacterial Cellulose Media

Stabilised rice bran was sourced from SunRice, Yanco, New South Wales, Australia. The bran stabilisation process, which involves heat treatment to deactivate lipase enzymes while preserving nutritional value, uses a proprietary and confidential commercial method. Cereal dust was obtained from Rex James Stockfeed Ltd., Nathalia, Victoria, Australia. Proximate analyses of both the rice bran and cereal dust substrates were conducted by certified commercial testing laboratories to determine their carbohydrate and protein content for media reformulation purposes. The results of these analyses are presented in Supplementary Table S1. This approach ensures the accuracy and reliability of the compositional data used in our study.

### 2.3. Reformulation of HS Culture Medium Using Agro Wastes for BC Production

Based on a proximate analysis, the standard HS bacterial cellulose production medium was reformulated by substituting key carbon and nitrogen sources. The reformulated media composition is presented in Supplementary Table S2.

### 2.4. Solid-State Fermentation of Rice Bran (RB) and Cereal Dust (CD)

The solid-state fermentation of rice bran and cereal dust was carried out using three fungal species: *Rhizopus oryzae* (RO) from NATURITAS, Barcelona, Spain; *Pleurotus osteratus* (PO) from Urban Spores, Queensland, Australia; and *Rhizopus oligosporus* (T) from Nourish Me Organics, Victoria, Australia. The pre-treatment of RB and CD using SSF was carried out in 5 L Erlenmeyer flasks for a better surface area and controlled aeration during the treatment. For each substrate, 200 g of material was evenly distributed at the bottom of the flask. The samples were moistened with distilled water prior to sterilisation. Each flask was inoculated with a fungal spore suspension containing 4.0 × 10^6^ spores medium, which was prepared using a haemocytometer [23]. The flasks were covered with sterile gauze to allow ventilation and incubated at 25 °C for 14 days in a Thermoline Refrigerated Incubator (Model Number—TRISLH-495-1-SD). After incubation, the fermented samples were sterilised at 121 °C for 16 min to decontaminate the fungal spores. The treated substrates were then oven-dried (LABEC) at 60 °C for 48 h until moisture-free, ground into a fine powder, and used as culture media components for BC production [13].

### 2.5. BC Production from Treated and Untreated Waste Substrates

Small-scale and large-scale experiments compared BC production using treated and untreated rice bran (RB) and cereal dust (CD) against standard HS medium. For small-scale trials, 50 mL tubes contained waste media formulations substituting HS carbon and nitrogen sources. SSF-treated and untreated substrates were used in equal amounts. The media were sterilised, adjusted to pH 6.2 (Mettler Toledo SevenEasy S20 pH Meter, Australia), and inoculated with *Novacetomonas* sp. (0.1 OD). The cultures were incubated statically at 28 °C for 14 days. BC pellicles were harvested, purified, and weighed after oven-drying and lyophilisation. On the other hand, large-scale production used 1L Schott bottles to increase the surface area, following the same protocol. This approach was based on studies showing BC yield is proportional to vessel surface area [24,25]. Inoculum size, pH, temperature, incubation period, and processing methods remained consistent with the small-scale trials.

### 2.6. Characterisation of BC Produced from SSF-Treated Substrates

Analytical techniques such as Fourier-transform infrared spectroscopy (FTIR), scanning electron microscopy (SEM), energy-dispersive X-ray spectroscopy (EDS), X-ray diffraction (XRD), thermogravimetric analysis (TGA), and derivative thermogravimetry (DTG) were used to study and evaluate the comparable properties of BC produced from SSF-treated waste media. The physico-chemical characterisations of these BC films were compared to the BC films generated from HS medium taken as a control, nata de coco as a commercially available BC control, and untreated BC samples (RB and CD). The comparable properties of these BC films generated from different waste media formulations were also studied based on two drying techniques: oven-drying (OD) in a hot air oven at 60 °C for 8 h and freeze-drying (FD) by lyophilising the samples at −80 °C for 6 h and moving them to freeze dryer (LABCONCO FreeZone 4.5 Plus, Australia) at a condenser temperature of −120 °C and vacuum pressure of 0.5 mbar until the weight remained constant. Following are the methods used for the preparation and analysis of BC films for each characterisation performed.

### 2.7. Scanning Electron Microscopy (SEM) and Energy-Dispersive X-ray Spectroscopy (EDS)

A field-emission scanning electron microscope (Zeiss Supra 40 VP, Jena, Germany) was used to observe the structural morphology of the ultrafine fibre network. BC pellicles obtained after the oven and freeze-drying were cut into small pieces and coated with a thin layer of gold onto the sample by a sputter coating device for 15 s, followed by the attachment of double-sided conducting carbon tape to the slide for better conductivity. The coated samples were viewed at different magnifications. Energy-dispersive X-ray spectroscopy (EDS) (Oxford Inca Energy 200, Thornleigh, Australia) was also performed simultaneously on the samples coated for SEM analysis.

### 2.8. Fourier-Transformed Infrared Spectroscopy (FTIR)

The infrared spectra of the BC were recorded using FTIR-VERTEX 70, Burker, Australia. BC samples were prepared as films to analyse, performing over the range of 400–4000 cm^−1^ with a resolution of 16 cm^−1^ and averaged over 200 scans. The spectrum line was baseline-corrected and optimised on the generated IR spectra using Quasar 1.7.0 and Origin 2023 software to compare and study the spectra of BC film samples from different reformulated media and drying methods.

### 2.9. X-ray Diffraction (XRD)

The bacterial cellulose crystal structure was analysed using an X-ray diffractometer (Bruker D8 Advance, Bruker, Australia) 5–80, 0.02, 0.5 s, 0 rpm, 10 × 10 mm variable slit, automatic air scatter (32 min). The oven- and freeze-dried samples of BC were prepared as thin and flat layers using glass slides and mounted on the sample stage. The samples were scanned at the rate of 0.5 s per step. The X-ray diffractogram was analysed and processed using Origin 2023 Peak Analyser Software.

The crystallinity index (CI) was calculated from the obtained diffractogram using the following equation [26]:


Crystallinity Index (CI) = (I_002_ − I_AM_/I_002_) × 100

The I_002_ represents the highest intensity of the 2θ peak, which is to say around 22.5° corresponds to the crystalline region, and I_AM_ represents the intensity of the peak situated between the (110) and (200) peaks, that is, 18° corresponds to the amorphous region.

### 2.10. Thermogravimetric Analysis (TGA)

The analysis of the phase transitions and decomposition behaviours of the BC films were performed using a Mettler Toledo TGA analyser, Melbourne, Australia. Around 20–30 mg of finely cut dried BC samples were used. The sample’s weight was continuously monitored while subjected to a controlled temperature increase from 30 °C to 800 °C.

## 3. Results

This study demonstrated the successful production of bacterial cellulose (BC) from rice bran and cereal dust pre-treated by SSF using *R. oryzae*, *P. osteratus*, and *R. oligosporus* (Figure 1). SSF pre-treatment enhanced the BC yield compared to untreated substrates, with rice bran fermented by *R. oligosporus* producing the highest yield. The BC produced from SSF-treated substrates showed a comparable nanostructure to that produced in standard HS medium and commercial nata de coco. Both the choice of fungal strain for SSF and the drying method (oven drying vs. freeze drying) significantly influenced the BC nanostructure, fibre diameter, and morphology. The physicochemical properties of BC, including crystallinity, thermal stability, and surface chemistry, were affected by the substrate type and processing conditions. Lastly, a cost analysis revealed that using SSF-treated agro-food wastes could reduce BC media costs by up to 90% compared to the standard HS medium. These results highlight the potential of SSF-treated agro-food wastes as sustainable and cost-effective substrates for BC production. The following subsections will discuss these findings in detail, including the characterisation of BC properties through various analytical techniques.

### 3.1. Solid-State Fermentation of Rice Bran and Cereal Dust

After 14 days of SSF, extensive fungal growth was observed on all samples (Supplementary Figure S1), with all three strains (*R. oryzae*, *P. osteratus*, and *R. oligosporus*) effectively colonising the rice bran (RB) and cereal dust (CD) substrates. These samples were autoclaved and decontaminated before harvesting the treated substrates. After the decontamination step, the samples were oven-dried and ground into a powder to mimic the substitutes for C and N sources in the HS medium. Visual inspection indicated successful bioconversion of the substrates by the fungal strains, as evidenced by the visible mycelial growth and changes in substrate texture (Supplementary Figure S1). The RB samples exhibited a more compact and darker appearance post-fermentation, while the CD samples showed a lighter, more friable texture. These observations suggest that SSF effectively altered the physicochemical properties of the substrates, improving nutrient accessibility for subsequent bacterial cellulose production. The use of different fungal strains resulted in subtle variations in the fermented substrates, which may influence their efficacy as culture media components.

The SSF process was completed without the addition of harsh chemicals or energy-intensive treatments, relying solely on the native enzyme systems of the fungal strains to modify the substrates. This approach aligns with green chemistry principles and potentially reduces processing costs. Other pre-treatments, such as enzymatic hydrolysis and alkali treatment, have also been investigated to improve the nutritional quality of rice bran. However, these methods are more expensive and often involve the use of harsh chemicals, making them less favourable compared to SSF [27].

### 3.2. Media Substitution Using SSF-Treated Rice Bran and Cereal Dust

**Figure 2:**
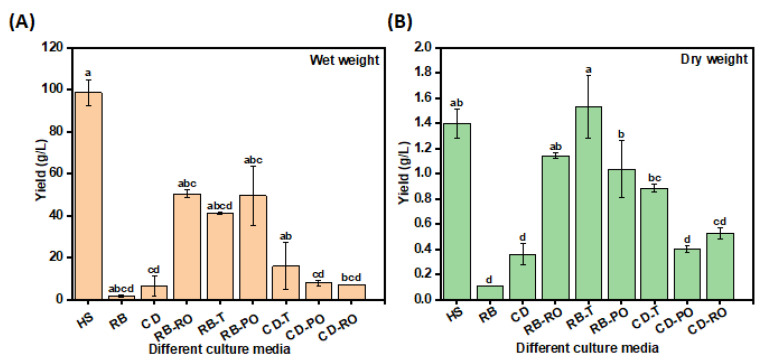
Large-scale BC production yield from untreated and treated waste media formulations. The (**A**) wet and (**B**) dry weight were determined on different waste formulations: Hestrin–Schramm (HS), rice bran (RB), cereal dust (CD), rice bran fermented with *R. oryzae* (RB-RO), rice bran fermented with *R. oligosporus* (RB-T), rice bran fermented with *P. osteratus* (RB-PO), cereal dust fermented with *R. oligosporus* (CD-T), cereal dust fermented with *P. osteratus* (CD-PO), cereal dust fermented with *R. oryzae* (CD-RO). The error bar represents the standard deviation. Different letters represent significant differences.

Substituting the carbon and nitrogen sources in the HS medium with SSF-treated rice bran (RB) and cereal dust (CD) significantly enhanced bacterial cellulose (BC) yields compared to the untreated substrates. This was demonstrated through both small-scale and large-scale experiments. Small-scale screening revealed that rice bran fermented with *Rhizopus oryzae* (RB-RO) yielded the highest BC (0.5 g/L), matching the conventional HS medium and surpassing untreated RB (Supplementary Figure S2). This improvement can be attributed to the action of fungal lignocellulolytic enzymes and proteases, which increased the bioavailability of nutrients. Large-scale experiments in 1L Schott bottles confirmed these findings, with rice bran fermented with *Rhizopus oligosporus* (RB-T) yielding the highest BC (1.5 ± 0.6 g/L dry weight), surpassing even the HS medium (Figure 2). This scale-up demonstrated the potential for industrial-scale production using SSF-treated agricultural wastes.

The superior performance in larger vessels aligns with previous studies reporting the influence of surface area to volume ratio on BC yield [25,28,29,30]. The wet weight BC yields obtained are comparable to or higher than those reported for other pre-treated agro-wastes, such as rice bark (2.4 g/L), oat hulls (2.2 g/L), wheat straw (8.3 g/L), and potato peel waste (4.7 g/L) [10,31,32]. These findings underscore the promise of combining SSF with the biosynthetic capabilities of *Novacetomonas* sp. for cost-effective and sustainable BC production from abundant waste streams.

### 3.3. Comparative Analyses of the BC Produced from SSF-Treated Rice Bran and Cereal Dust

#### 3.3.1. Scanning Electron Microscopy (SEM)

**Figure 3:**
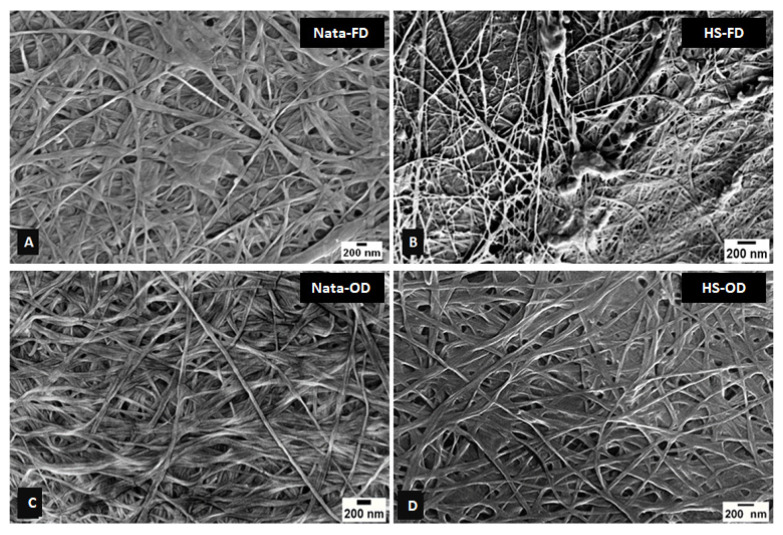
SEM images of commercially available nata de coco cellulose and cellulose produced from *Novacetomonas* sp. in HS culture medium. The (**A**,**C**) freeze-(FD) and oven-dried (OD) nata de coco, (**B**,**D**) freeze-(FD) and oven-dried (OD) cellulose from *Novacetomonas* sp. (scale bar—200 nm).

SEM analysis revealed distinct differences in BC nanostructure based on both the substrate source and drying method. Figure 3 compares the nanostructure of BC produced in HS medium with commercially available nata de coco, while Figure 3 and Supplementary Figure S3 show BC derived from SSF-treated cereal dust and rice bran, respectively.

Across all samples, freeze-dried (FD) BC exhibited a more open, porous structure with visible spaces between fibre bundles, while oven-dried (OD) samples displayed a dense, interconnected network of nanofibers (Figure 3). This trend was consistent for BC produced from HS medium, nata de coco, and SSF-treated agricultural wastes. The effect of the substrate source on the BC nanostructure was evident in the comparison of cereal dust- and rice bran-derived BC (Figure 3 and Supplementary Figure S3). BC from CD fermented with *Pleurotus osteratus* showed a particularly dense and interconnected network, similar to that observed by Mohammadkazemi et al. (2015) in BC produced from mannitol and sucrose media [33]. Samples pre-treated with *Rhizopus oryzae* and *Rhizopus oligosporus* exhibited slightly more varied fibre arrangements.

**Figure 4:**
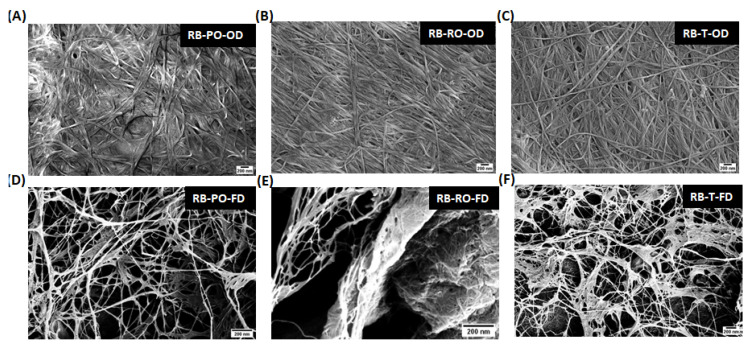
SEM micrographs of BC samples produced from fermented rice bran, using *P. osteratus*, *R. oryzae*, and *R. oligosporus* for SSF. The (**A**) oven-(RB-PO-OD) and (**D**) freeze-dried (RB-PO-FD) samples using *P. osteratus*, the (**B**) oven-(RB-RO-OD) and (**E**) freeze-dried (RB-RO-FD) samples using *R. oryzae*, and the (**C**) oven-(RB-T-OD) and (**F**) freeze-dried (RB-T-FD) samples using *R. oligosporus* (scale bar—200 nm).

**Figure 5:**
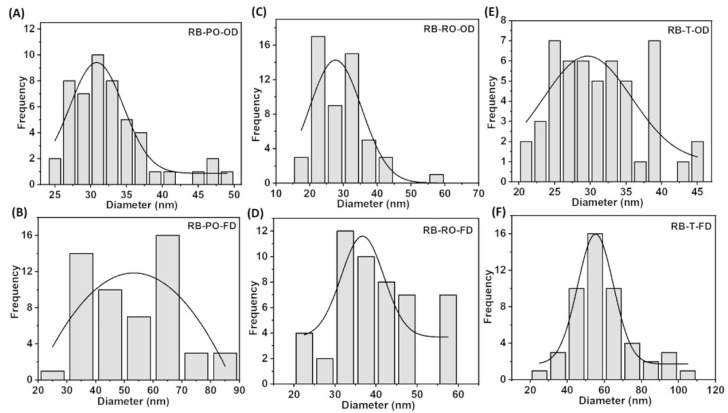
Fibre diameter graphs of BC produced from fermented rice bran, using *P. osteratus*, *R. oryzae*, and *R. oligosporus* for SSF. The (**A**) oven-(RB-PO-OD) and (**B**) freeze-dried (RB-PO-FD) samples using *P. osteratus*, the (**C**) oven-(RB-RO-OD) and (**D**) freeze-dried (RB-RO-FD) samples from *R. oryzae*, and the (**E**) oven-(RB-T-OD) and (**F**) freeze-dried (RB-T-FD) samples using *R. oligosporus*. The curve represents the mean value of the fibre diameter.

A fibre diameter analysis (Figure 4 for cereal dust and Supplementary Figure S4 for rice bran) revealed that OD samples consistently produced smaller average fibre diameters (around 30 nm) compared to FD samples (40–50 nm for rice bran, 30–80 nm for cereal dust). This trend aligns with observations by Du et al. (2018), confirming the influence of drying methods on BC nanostructure across various production conditions [34]. Interestingly, the effect of the fungal strain on fibre diameter was less pronounced in the RB samples compared to cereal dust, suggesting that substrate composition may interact with fungal pre-treatment to influence the final BC nanostructure (Figure 5).

These results collectively emphasise the importance of both substrate choice and processing conditions in determining BC nanostructure. The ability to fine-tune fibre dimensions and network morphology through substrate selection, fungal pre-treatment, and drying method offers a versatile toolkit for engineering BC with specific properties suited to various applications [33,34,35,36,37,38,39].

#### 3.3.2. Energy-Dispersive X-ray Spectroscopy (EDS) Analysis

EDS analysis revealed that BC produced from SSF-treated rice bran and cereal dust primarily consisted of carbon and oxygen, confirming the preservation of cellulosic composition (Table 1). BC from SSF-treated rice bran showed a higher carbon content (65–69%) compared to cereal dust (53–64%), while cereal dust-derived BC exhibited a higher oxygen content (35–46% vs. 31–35% in rice bran-derived BC). These differences were consistent across oven-dried and freeze-dried samples. The higher oxygen content in cereal dust-derived BC suggests a greater presence of O-containing functional groups, potentially influencing fibre interactions and network compactness. The observed variations in C/O ratios between substrates and fungal treatments indicate that the choice of waste substrate and SSF conditions can influence the BC surface chemistry. These findings align with previous studies showing that BC composition can be affected by bacterial strain, growth conditions, and carbon source [40,41]. Importantly, EDS analysis confirmed that SSF pre-treatment did not introduce detectable contaminants, demonstrating the potential of this method for producing pure BC from agricultural wastes.

#### 3.3.3. Fourier-Transformed Infrared Spectroscope (FTIR)

**Figure 6:**
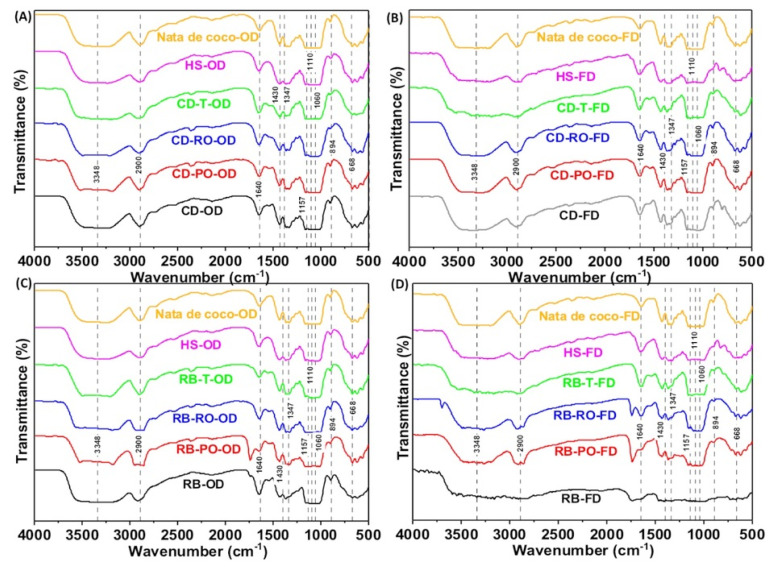
FTIR spectra of oven- and freeze-dried BC synthesised from SSF-treated cereal dust using *P. osteratus*, *R. oryzae,* and *R. oligosporus* during SSF. BC synthesised from SSF-treated cereal dust with (**A**,**B**) *P. osteratus* (CD-PO-OD/FD), *R. oryzae* (CD-RO-OD/FD), and *R. oligosporus* (CD-T-OD/FD). BC synthesised from SSF-treated rice bran with (**C**,**D**) *P. osteratus* (RB-PO-OD/FD), *R. oryzae* (RB-RO-OD/FD), and *R. oligosporus* (RB-T-OD/FD).

FTIR analysis of BC produced from SSF-treated rice bran and cereal dust revealed characteristic cellulose peaks, confirming the preservation of a cellulose type I structure (Figure 5). The key spectral peaks included O-H stretching (3300 cm^−1^), C-H stretching (2900 cm^−1^), adsorbed water (1640 cm^−1^), CH_2_ bending (crystallinity band, 1427 cm^−1^), C-H bending (1350 cm^−1^), C-O-C stretching (1160–1110 cm^−1^), and C-O stretching (1060 cm^−1^). These peaks are consistent with those reported for BC produced from conventional media [42,43]. Some rice bran-derived samples (RB-FD, RB-PO-OD, RB-PO-FD, RB-RO-FD) showed an additional peak around 1700 cm^−1^, potentially indicating a slight oxidation of surface hydroxyls. In general, the FTIR spectra confirm that BC produced from SSF-treated agricultural wastes maintains the essential chemical structure of pure cellulose, with minor variations that may offer opportunities for tailoring physicochemical properties. It is worth noting that no characteristic lignin peaks were observed in the FTIR spectra, confirming the purity of the bacterial cellulose produced. This is consistent with the known ability of our *Novacetimonas* sp. strain to synthesise pure cellulose. These findings align with previous studies using other waste substrates for BC production [31,44,45] demonstrating the versatility of RB and CD wastes as feedstocks for BC synthesis (Figure 6).

#### 3.3.4. X-ray Diffraction (XRD)

**Figure 7:**
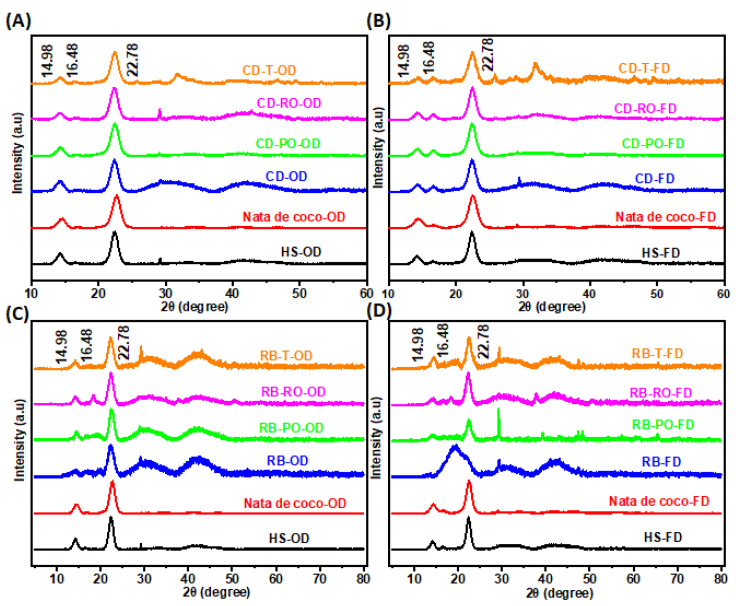
X-ray diffraction patterns of BC synthesised from SSF-treated cereal dust and rice bran. BC samples from (**A**,**B**) cereal dust (CD) and (**C**,**D**) rice bran (RB) using *P. osteratus* (PO-OD/FD), *R. oryzae* (RO-OD/FD), and *R. oligosporus* (T-OD/FD) compared to nata de coco and HS medium, oven-dried (OD) and freeze-dried (FD).

XRD patterns of BC produced from SSF-treated RB and CD revealed characteristic peaks at 2θ = 14.9°, 16.5°, and 22.8°, corresponding to the typical cellulose I structure (Figure 6A–D) [46,47,48]. These peaks were consistent across all samples, including the nata de coco and HS medium controls, confirming the preservation of BC’s crystalline structure (Figure 7). However, some subtle differences were observed between samples. The BC from SSF-treated substrates showed additional broad peaks centred at 30° and between 40 and 50°, not present in the control samples. The freeze-dried rice bran samples exhibited a significant peak at 30°, corresponding to the (122) plane, which was absent in most oven-dried samples. Lastly, the freeze-dried rice bran samples generally showed broader peaks, indicating a lower crystallinity compared to the oven-dried samples. These variations suggest that both the SSF pre-treatment and drying method influence the crystalline structure of the produced BC. The presence of additional peaks and changes in peak intensity indicate potential modifications in cellulose arrangement and crystallinity, which could affect the material properties of the resulting BC. A similar study used heat-pre-treated molasses culture media for BC production and made similar observations in the XRD peaks of the BC films generated from waste, which had peaks identical to the HS medium and showed the characteristic peaks of the crystalline form of cellulose I [1].

The crystallinity index (CI) of the BC samples is presented in Supplementary Table S3, calculated based on the intensity of peaks at 2θ = 22.5° and 18.5°, corresponding to the (110) and (200) planes of the crystalline form of cellulose I [1]. It was revealed that the BC from cereal dust showed a high CI (94–99%), with oven-dried samples reaching ~99%. In contrast, the BC from rice bran exhibited a lower CI, particularly in freeze-dried samples (69–79%). In terms of drying, oven-dried samples consistently showed a higher CI than freeze-dried samples for both substrates. Lastly, the SSF treatment with different fungi had a minimal impact on the CI for cereal dust but affected rice bran samples more noticeably. These results align with the XRD patterns, where sharper, more defined peaks correlate with a higher crystallinity. The high CI of cereal dust-derived BC suggests a well-ordered cellulose structure, while the lower CI in rice bran-derived BC, especially freeze-dried samples, indicates increased amorphous regions. The variations in CI between substrates and drying methods demonstrate the potential to modulate BC crystallinity through feedstock selection and processing conditions. Higher crystallinity generally correlates with improved mechanical properties, while lower crystallinity may enhance properties such as swelling and drug-loading capacity. These findings suggest opportunities for tailoring BC properties for specific applications by controlling its crystallinity through waste media substrate choice and processing methods.

#### 3.3.5. Thermogravimetric Analysis (TGA)

**Figure 8:**
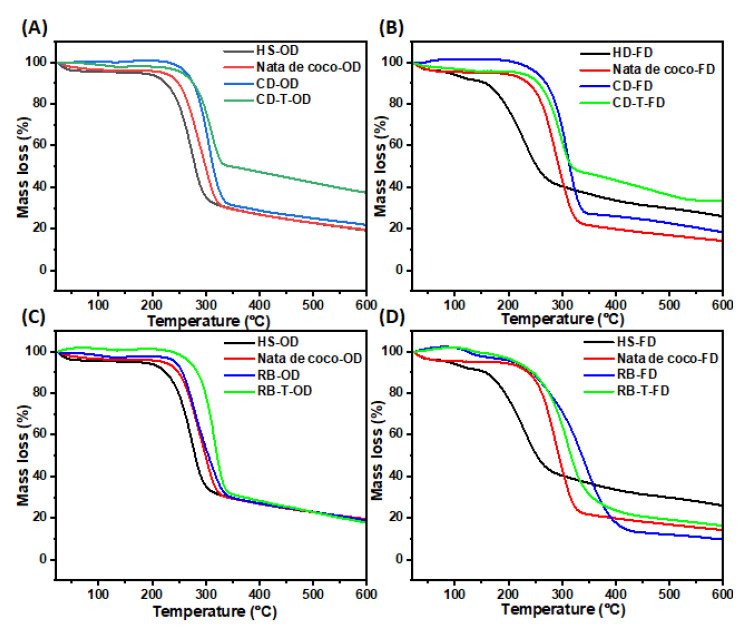
Thermogravimetric analysis of BC samples produced in untreated and treated cereal dust and rice bran using *R. oligosporus* (T) for SSF. (**A**) BC samples produced using cereal dust (CD)*,* oven-(OD) and (**B**) freeze-dried (FD). (**C**) BC samples produced using rice bran (RB), oven-(OD) and (**D**) and freeze-dried (FD).

The thermogravimetric analysis of BC samples produced from untreated and SSF-treated cereal dust and rice bran revealed distinct thermal degradation profiles (Figure 8). The initial mass loss for most samples occurred around 250 °C, indicating the onset of thermal degradation. The BC from SSF-treated cereal dust (CD-T-OD) showed the highest thermal stability, with only ~60% mass loss at 600 °C (Figure 8A). Additionally, the freeze-dried samples generally exhibited lower thermal stability compared to their oven-dried counterparts. Moreover, the BC from SSF-treated rice bran (RB-T-OD) maintained its structure up to 290 °C, showing improved thermal stability compared to the untreated samples (Figure 8C). In contrast, HS medium-derived BC, particularly freeze-dried (HS-FD), showed the lowest thermal stability, with degradation starting at 150 °C. It was also observed that the SSF treatment generally improved the thermal stability of BC from both RB and CD media compared to the untreated samples and control BC (nata de coco and HS medium). These results suggest that both the substrate type and SSF treatment influence the thermal properties of the resulting BC. The enhanced thermal stability of SSF-treated samples indicates potential improvements in BC structure and composition, which could be beneficial for applications requiring heat resistance.

**Figure 9:**
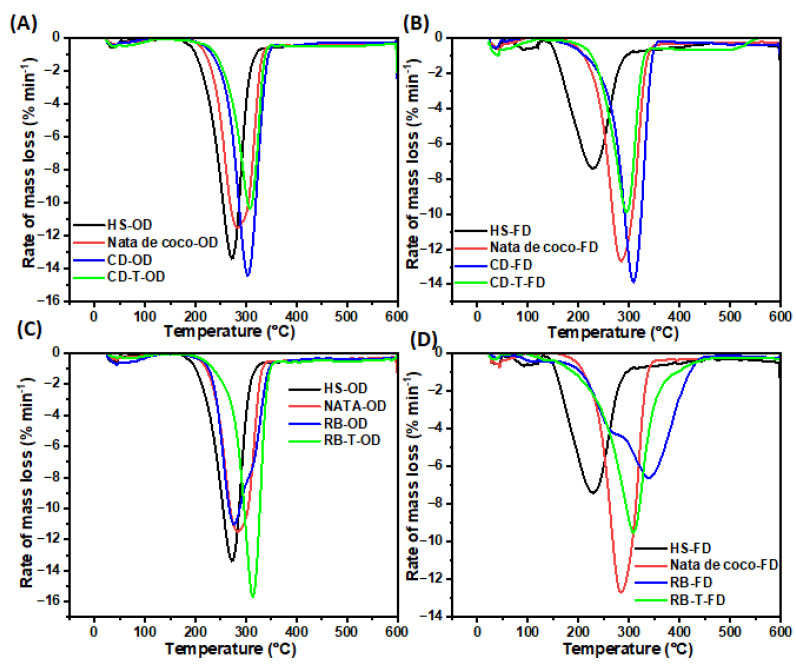
Derivative thermogravimetry (DTG) analysis of BC samples produced in untreated and treated cereal dust and rice bran with using *R. oligosporus* (T) for SSF. (**A**) BC samples produced using cereal dust (CD)*,* oven-(OD) and (**B**) freeze-dried (FD); (**C**) BC samples produced using rice bran (RB), oven-(OD) and (**D**) freeze-dried (FD).

Derivative thermogravimetry (DTG) analyses revealed distinct thermal degradation profiles for BC samples produced from SSF-treated and untreated cereal dust and rice bran (Figure 9). The SSF-treated cereal dust BC (CD-T-OD) showed an improved thermal stability with a lower mass loss rate (10%) at 300 °C compared to untreated samples (14%) (Figure 9A). The freeze-dried SSF-treated cereal dust BC (CD-T-FD) exhibited the lowest mass loss rate (9.8%) at 290 °C among the cereal dust samples (Figure 9B). The SSF-treated rice bran BC (RB-T-OD) showed a higher mass loss rate (16%) at a higher temperature (320 °C) compared to untreated samples (11% at 280 °C) (Figure 9C). Lastly, the freeze-dried rice bran BC (RB-FD) demonstrated the lowest mass loss rate (7%) at 320 °C among all samples (Figure 9D). These results indicate that SSF treatment generally enhances the thermal stability of BC, with the effect more pronounced in cereal dust samples. The improved thermal properties likely result from increased crystallinity and better-organised cellulose structures, as suggested by XRD analysis (Figure 6 and Table S3). Our findings align with the previous studies, which showed enhanced thermal stability in BC produced from agricultural wastes [33]. The enhanced thermal stability of SSF-treated BC samples suggests potential applications in areas requiring heat-resistant materials, such as biomedical devices or electronics. Furthermore, the ability to modulate thermal properties through substrate selection and processing conditions offers opportunities for tailoring BC for specific applications.

#### 3.3.6. Cost Analysis of Growth Media Input

A detailed comparison of the production costs of bacterial cellulose (BC) based on the input resources used for the culture media has shown significant cost savings when alternative SSF-treated substrates are employed (Table 2). Replacing carbon and nitrogen sources in HS medium with unfermented RB and CD lowers the cost by up to 40%. Meanwhile, using the SSF process to ferment these wastes has not only increased the yield of BC but also reduced the cost of media by around 90%. Table 2 shows the cost analysis of the conventional HS medium with unfermented and fermented RB and CD.

The BC (dry weight) produced from SSF-treated RB and CD costs around AUD 2.51 per gram compared to untreated RB and CD, which is AUD 13.51 per gram. In contrast, BC produced using standard HS medium costs around AUD 17.08 per gram. For reference, the approximate cost of commercially available BC in the market is around AUD 29.00 per gram. This substantial decrease in cost is attributed to the dual benefits of using low-cost, abundant waste materials and enhancing the process efficiency of SSF. The cost reduction, coupled with the improved BC yield, suggests that SSF-treated agricultural wastes could be a highly economical and sustainable approach for large-scale BC production. These findings have significant implications for industrial-scale BC production, potentially lowering barriers to entry for BC-based products in various sectors. Moreover, this approach aligns with circular economy principles by valorising agricultural waste streams. Future studies should focus on scaling up this process and conducting a full life-cycle assessment to comprehensively evaluate its environmental and economic impacts.

## 4. Discussion

This study demonstrates the successful production of bacterial cellulose (BC) from rice bran and cereal dust pre-treated by SSF, representing a significant step towards sustainable and cost-effective BC production. The use of this abundant agricultural waste addresses two critical challenges: the high cost of conventional BC production media and the environmental impact of agro-industrial waste. SSF pre-treatment effectively enhanced the bioavailability of nutrients in rice bran and cereal dust, resulting in improved BC yields compared to untreated substrates. Notably, rice bran fermented with *Rhizopus oligosporus* produced the highest BC yield (1.52 g/L dry weight), surpassing even the standard HS medium. This finding aligns with previous studies showing the potential of SSF to unlock nutrients from complex biomass and extends this approach to BC production [13,19]. On the other hand, it was also shown that the surface area of a vessel is an important parameter, especially in a static cultivation setting, as it impacts the oxygen transport in the medium for BC production [25,30].

A comprehensive physicochemical characterisation revealed that BC produced from SSF-treated substrates maintains the fundamental nanostructure and chemical identity of cellulose, with some variations that could be advantageous for specific applications. SEM analysis showed that both the choice of fungal strain for SSF and the drying method (oven drying vs. freeze drying) significantly influenced the BC nanostructure, fibre diameter, and morphology. These structural differences could be leveraged to tailor BC properties for various applications, from tissue engineering scaffolds to filtration membranes [49]. FTIR and XRD analyses confirmed the preservation of a cellulose I structure in BC from SSF-treated substrates, with some samples showing additional peaks that suggest slight modifications to surface chemistry or crystallinity. These subtle changes could potentially enhance specific properties, such as thermal stability or reactivity, without compromising the essential cellulose structure. The observed variations in crystallinity index between substrates and drying methods demonstrate the potential to modulate BC properties through feedstock selection and processing conditions. On the other hand, thermogravimetric analysis revealed that SSF treatment generally improved the thermal stability of BC from both rice bran and cereal dust media. This enhanced thermal resistance could expand the potential applications of BC to areas requiring heat-stable materials, such as biomedical devices or electronics components [33].

Perhaps most significantly, the cost analysis revealed that using SSF-treated agro-food wastes could reduce BC media costs by up to 90% compared to the standard HS medium. This dramatic cost reduction, coupled with the valorisation of agricultural waste streams, presents a compelling case for the industrial adoption of this approach, aligning with current trends in circular bioeconomy strategies. The potential for significant cost savings while maintaining or improving BC quality could be advantageous for the large-scale production of this versatile biopolymer.

The findings of this study have broad implications for both sustainable material production and waste management in the agro-food industry. By demonstrating the feasibility of using minimally processed agricultural wastes for high-value biopolymer production, this research contributes to the growing body of work on circular bioeconomy strategies. The approach developed here could be extended to other types of agro-industrial wastes, potentially revolutionising the production of bio-based materials while simultaneously addressing waste management challenges. Moreover, the ability to modulate BC properties through substrate selection, SSF conditions, and drying methods offers new avenues for tailoring this material for specific applications. This versatility could expand the use of BC across various sectors, from biomedical applications to advanced materials for electronics and environmental remediation [50]. Hence, this study provides a proof-of-concept for the sustainable valorisation of agro-food wastes into high-value BC biomaterials. These improvements in yield, cost-effectiveness, and potential for property customisation pave the way for scaled-up, environmentally friendly BC production processes.

Future research should focus on optimising SSF conditions for different waste streams, exploring the full range of attainable BC properties, and developing specific applications that leverage the unique characteristics of waste-derived BC. Life-cycle assessments and scaled-up production studies are needed to fully evaluate the environmental and economic impacts of this approach. Hence, future work should include a comprehensive mass balance study of the entire process, from SSF pre-treatment to BC production. This will involve a detailed quantification of all input materials (cereal wastes, fungi, bacteria) and output streams (BC, residual biomass, metabolites) at each stage of the process. Such a study will provide crucial insights into the process efficiency, nutrient utilisation, and potential for further optimisation. Additionally, it will allow for a more accurate assessment of the environmental impact and sustainability of this BC production method.

## 5. Conclusions

The use of solid-state fermentation with fungi has proven to be highly effective in improving the digestibility and bioavailability of agricultural waste material, which in turn has led to a significant increase in bacterial cellulose production up to double the usual yield. Specific enzymes in this process have led to the breakdown of the complex structures within these waste materials, releasing essential nutrients without damaging the fundamental nanostructure or chemical properties of cellulose. Additionally, the drying process used in this method plays a crucial role in controlling the final structure of BC, allowing the precise customisation of the material’s properties. This includes adjusting features such as the width of the nanofibers and the size of the pores, which can be tailored to meet biofunctional needs. A detailed spectroscopic, microscopic, and thermal analysis confirmed the synthesis of signature bacterial cellulose I nanoparticles with commercially comparable properties. Materials characterisation revealed tunability in the thermal stability, surface traits, and nanostructure based on nutritional and drying variations. A cost analysis based on the resources used for the culture medium shows that producing BC from fermented agricultural by-products like rice bran and cereal dust is nearly 90% cheaper than using the traditional Hestrin–Schramm medium. Hence, this research demonstrates a new, integrated approach to converting cereal crop waste into valuable cellulose, offering a solution that is both economically viable and environmentally sustainable, while also allowing for the customisation of the final product’s properties.

## Figures and Tables

**Table 1:** Elemental composition of C and O in BC produced from fermented rice bran and cereal dust in OD and FD samples.

Samples	Oven-Dried		Freeze-Dried	
	C	O	C	O
Rice bran	68.0%	32.0%	75.1%	24.9%
Rice bran *(Pleurotus osteratus*)	68.7%	31.3%	63.7%	36.3%
Rice bran (*Rhizopus oryzae*)	67.0%	33.0%	57.6%	42.4%
Rice bran (*Rhizopus oligosporus*)	65.2%	34.8%	59.6%	40.4%
Cereal dust	65.4%	34.6%	59.8%	40.2%
Cereal dust (*Pleurotus osteratus*)	64.3%	35.7%	57.1%	42.9%
Cereal dust (*Rhizopus oryzae*)	63.2%	36.8%	53.8%	46.2%
Cereal dust (*Rhizopus oligosporus*)	59.9%	40.1%	53.8%	46.2%

**Table 2:** Cost analysis of bacterial cellulose produced from HS medium vs. unfermented and fermented rice bran (RB) and cereal dust (CD) in g/L.

		Price		Hestrin-Schramm Media/L		Rice Bran/L		Cereal Dust/L		SSF-Treated RB/L		SSF-Treated CD/L	
Media Composition	Unit	AU $/Unit	AU $/g	Quantity (g)	Cost ($/g)	Quantity (g)	Cost ($/g)	Quantity (g)	Cost ($/g)	Quantity (g)	Cost ($/g)	Quantity (g)	Cost ($/g)
Glucose	1 kg	63.5	0.06	20	1.2	0	0	0	0	0	0	0	0
Yeast extract	1 kg	360	0.36	5	1.8	0	0	0	0	0	0	0	0
Peptone	500 g	210	0.42	5	2.1	0	0	0	0	0	0	0	0
Di-sodium hydrogen phosphate	500 g	93.13	0.19	2.7	0.51	2.7	0.51	0.51	0.51	2.7	0.51	2.7	0.51
Citric acid monohydrate	1 kg	185	0.18	1.85	0.33	1.85	1.85	1.85	1.85	1.85	1.85	1.85	1.85
Magnesium sulphate heptahydrate	1 kg	287	0.28	0.5	0.14	0.5	0.15	0.15	0.15	0.5	0.15	0.5	0.15
Ethanol	1 L	117	0.11	100 mL	11	100	11	0	11	0	0	0	0
Rice bran	10 kg	0	0	0	0	76.9	0	0	0	76.9	0	0	0
Cereal Dust	5 kg	0	0	0	0	0	0	58.5	0	0	0	58.5	0
Total cost					**17.08**		**13.51**		**13.51**		**2.51**		**2.51**

## Data Availability

The raw data supporting the conclusions of this article will be made available by the authors on request. The 16S rRNA data for the molecular identification of the isolate are available at the NCBI as Genbank PP421219.1.

## Supplementary Materials

The following supporting information can be downloaded at: https://www.mdpi.com/article/10.3390/foods13193052/s1, Figure S1: Solid-state fermentation set up for rice bran (RB) and cereal dust (CD) in 5L Erlenmeyer flasks; Figure S2: Small-scale BC production yield from untreated and treated waste media formulations, Hestrin-Schramm (H-S), rice bran (RB), cereal dust (CD), rice bran fermented with *Rhizopus oryzae* (RB-RO), rice bran fermented with *Rhizopus oligosporus* (RB-T), rice bran fermented with *Pleurotus osteratus* (RB-PO), cereal dust fermented with *Rhizopus oligosporus* (CD-T), cereal dust fermented with *Pleurotus osteratus* (CD-PO), cereal dust fermented with *Rhizopus oryzae* (CD-RO) showing (A) wet and (B) dry weight yield (g/L). The error bar represents standard deviation. Different letters represent significant differences; Figure S3: SEM micrographs of BC produced from fermented cereal dust, with *Pleurotus osteratus*, oven-dried (A) (CD-PO-OD), freeze-dried (D) (CD-PO-FD), *Rhizopus oryzae* oven-dried (B) (CD-RO-OD), freeze-dried (E) (CD-RO-FD), *Rhizopus oligosporus* oven dried (C) (CD-T-OD), freeze-dried (F) (CD-T-FD) (scale bar- 200 nm); Figure S4: Fibre diameter graphs of BC produced from fermented cereal dust, with *Pleurotus osteratus*, oven-dried (A) (CD-PO-OD), freeze-dried (D) (CD-PO-FD), *Rhizopus oryzae* oven-dried (B) (CD-RO-OD), freeze-dried (E) (CD-RO-FD), *Rhizopus oligosporus* oven dried (C) (CD-T-OD), freeze-dried (F) (CD-T-FD). The curve represents the mean value of the fibre diameter. Table S1: Total carbohydrate and protein content of wastes used for formulating the waste media for BC production; Table S2: Reformulated media compositions using rice bran and cereal dust (untreated/treated) compared to HS media used for BC production; Table S3: The crystallinity index of the oven and freeze-dried samples of unfermented and fermented rice bran and cereal dust.
