# Supplementary material for: Solid-State Fermentation of Cereal Waste Improves the Bioavailability and Yield of Bacterial Cellulose Production by a Novacetimonas sp. Isolate
Source: Foods. 2024 Sep 25;13(19):3052. doi: 10.3390/foods13193052 (PMC11475563; doi:10.3390/foods13193052)
Supplement: Supplementary file 1 [file foods-13-03052-s001.zip › foods-3123843-supplementary.pdf]

SUPPLEMENTARY INFORMATION

Supplementary Tables

Supplementary Table S1. Total carbohydrate and protein content of wastes used for formulating the waste media for BC production.

Types of Wastes	Total Carbohydrate Content	Total Protein Content
Cereal Dust	44.6%	17.1%
Stabilised Rice Bran	23.0%	13.0%

Supplementary Table S2. Reformulated media compositions using rice bran and cereal dust (untreated/treated) compared to HS media used for BC production.

Media Component	HS Media (g/L)	Rice Bran Media (g/L)	Ad. Cereal Dust Media (g/L)	Cereal Dust Media (g/L)
Glucose	20.00	20.00	0.00	0.00
Yeast Extract	5.00	0.00	1.16	0.00
Peptone	5.00	0.00	1.16	0.00
Citric acid monohydrate	1.85	1.85	1.85	1.85
Magnesium sulphate heptahydrate	0.50	0.50	0.50	0.50
Disodium hydrogen phosphate	2.70	2.70	2.70	2.70
Rice Bran	0.00	76.90	0.00	0.00
Cereal Dust	0.00	0.00	44.9	58.50

Supplementary Table S3. The crystallinity index of the oven and freeze-dried samples of unfermented and fermented rice bran and cereal dust.

	Crystallinity Index	
**Bacterial cellulose samples**	**Oven-dried**	**Freeze-dried**
Rice bran	94.30%	-
Rice bran (*Pleurotus osteratus*)	83.80%	69.30%
Rice bran (*Rhizopus Oryzae*)	72.00%	72%
Rice bran (*Rhizopus oligosporus*)	95.20%	79.00%
Cereal Dust	98.80%	99.65%
Cereal Dust (*Pleurotus osteratus*)	98.60%	97.40%
Cereal dust (*Rhizopus oryzae)*	99.80%	96.20%
Cereal dust (*Rhizopus oligosporus*)	94.90%	89.80%

Supplementary Figures

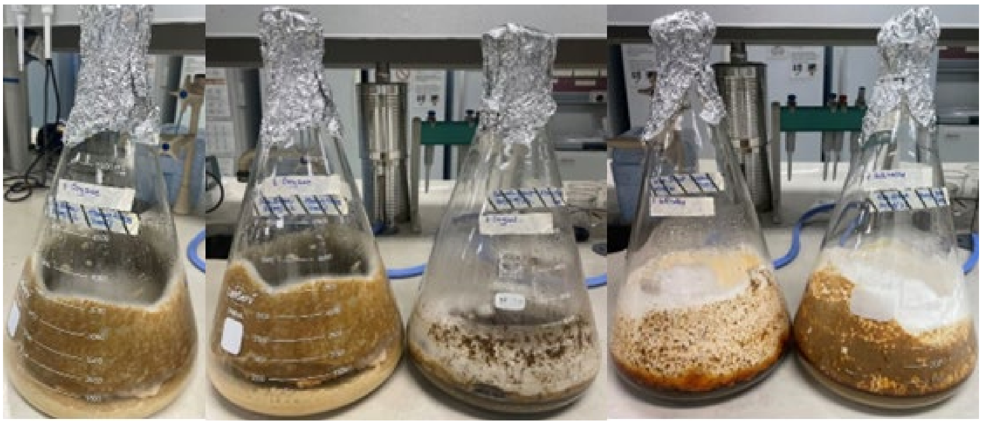

Supplementary Figure S1. Solid-state fermentation set up for rice bran (RB) and cereal dust (CD) in 5L Erlenmeyer flasks.

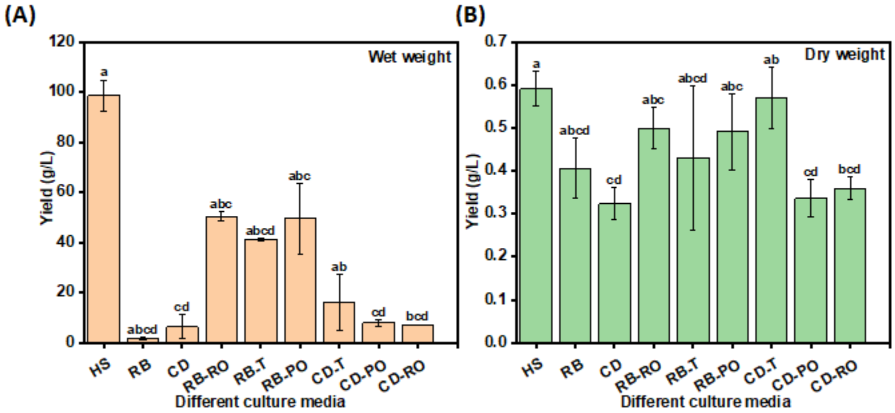

Supplementary Figure S2. Small-scale BC production yield from untreated and treated waste media formulations, Hestrin-Schramm (H-S), rice bran (RB), cereal dust (CD), rice bran fermented with *Rhizopus oryzae* (RB-RO), rice bran fermented with *Rhizopus oligosporus* (RB-T), rice bran fermented with *Pleurotus osteratus* (RB-PO), cereal dust fermented with *Rhizopus oligosporus* (CD-T), cereal dust fermented with *Pleurotus osteratus* (CD-PO), cereal dust fermented with *Rhizopus oryzae* (CD-RO) showing (A) wet and (B) dry weight yield (g/L). The error bar represents standard deviation. Different letters represent significant differences.

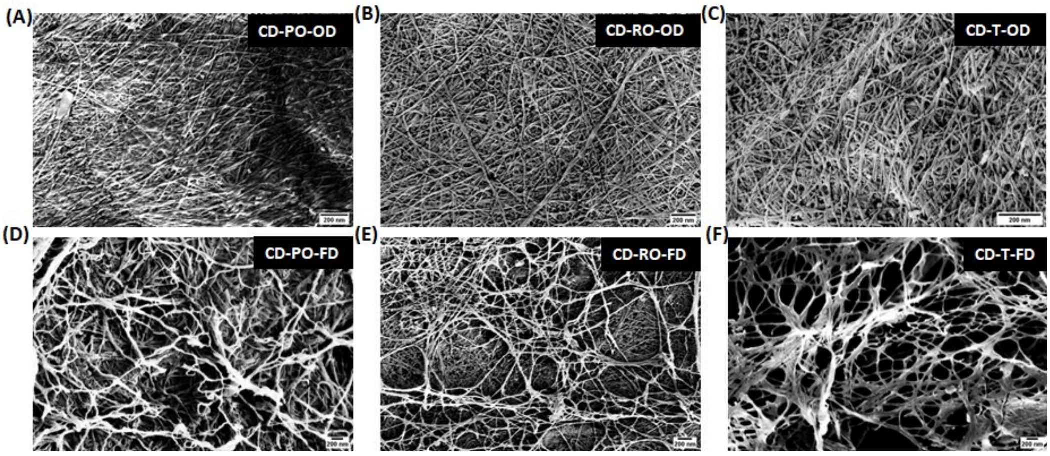

Supplementary Figure S3. SEM micrographs of BC produced from fermented cereal dust (CD), using *Pleurotus osteratus*, (A) oven- (CD-PO-OD) and (D) freeze-dried (CD-PO-FD), using *Rhizopus oryzae* (B) oven- (CD-RO-OD)

and (E) freeze-dried (CD-RO-FD), and using *Rhizopus oligosporus* (C) oven- (CD-T-OD) and freeze-dried (F) (CD-T-FD), using scale bar equal to 200 nm.

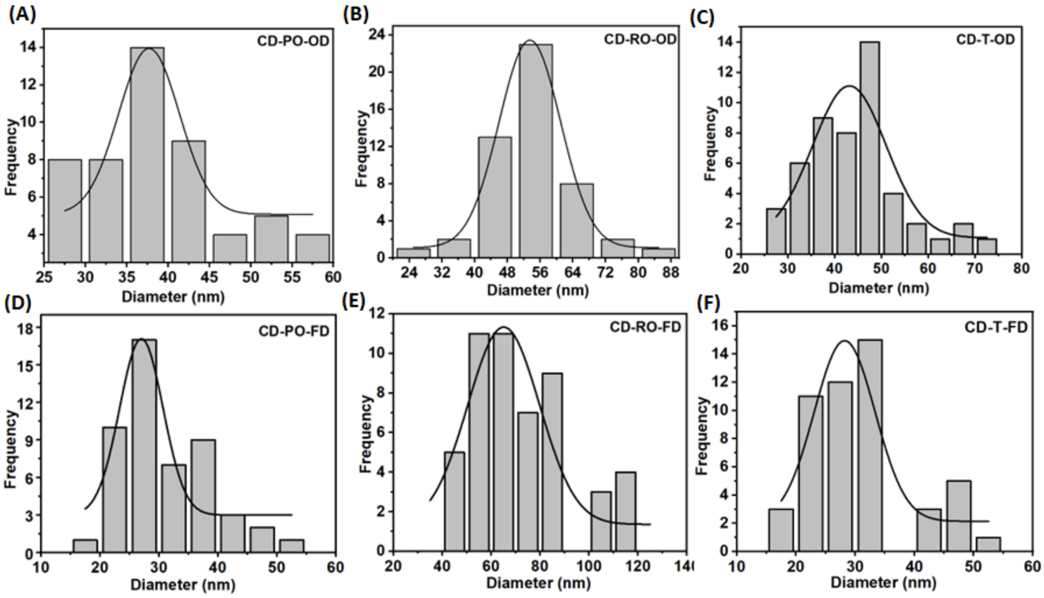

Supplementary Figure S4. Fibre diameter graphs of BC produced from fermented cereal dust, using *Pleurotus osteratus* (A) oven- (CD-PO-OD) and (D) freeze-dried (CD-PO-FD), using *Rhizopus oryzae* (B) oven- (CD-RO-OD) and (E) freeze-dried (CD-RO-FD), and using *Rhizopus oligosporus* (C) oven- (CD-T-OD) and (F) freeze-dried (CD-T-FD). The curve represents the mean value of the fibre diameter.
